# Not the Master of Your Volitional Mind? The Roles of the Right Medial Prefrontal Cortex and Personality Traits in Unconscious Introjections Versus Self-Chosen Goals

**DOI:** 10.3389/fpsyg.2022.740925

**Published:** 2022-04-29

**Authors:** Markus Quirin, André Kerber, Ekkehard Küstermann, Elise L. Radtke, Miguel Kazén, Carsten Konrad, Nicola Baumann, Richard M. Ryan, Michael Ennis, Julius Kuhl

**Affiliations:** ^1^School of Management, Technical University of Munich, München, Germany; ^2^Department of Psychology, PFH Göttingen, Göttingen, Germany; ^3^Department of Education and Psychology, Freie Universität Berlin, Berlin, Germany; ^4^Department of Neuropsychology and Behavioral Neurobiology, University of Bremen, Bremen, Germany; ^5^Institute of Psychology, Osnabrück University, Osnabrück, Germany; ^6^Department of Medicine, Philipps-Universität Marburg, Marburg, Germany; ^7^Department of Psychology, Trier University, Trier, Germany; ^8^Institute for Positive Psychology and Education, Australian Catholic University, Sydney, NSW, Australia; ^9^Department of Clinical and Social Sciences, University of Rochester, Rochester, NY, United States; ^10^Department of Psychology, California State University at Chico, Chico, CA, United States

**Keywords:** introjection, self-infiltration, self-determination, rumination, action-state orientation, emotional self-awareness, extraversion, neuroticism

## Abstract

Humans are unconditionally confronted with social expectations and norms, up to a degree that they, or some of them, have a hard time recognizing what they actually want. This renders them susceptible for introjection, that is, to unwittingly or “unconsciously” mistake social expectations for self-chosen goals. Such introjections compromise an individual’s autonomy and mental health and have been shown to be more prevalent in individuals with rumination tendencies and low emotional self-awareness. In this brain imaging study, we draw on a source memory task and found that introjections, as indicated by imposed tasks that are falsely recognized as self-chosen, involved the bilateral medial prefrontal cortex (MPFC) and the dorsal anterior cingulate cortex (ACC). Notably, reduced right MPFC activation within this condition correlated with trait scores of ruminations and reduced emotional self-awareness, but also introversion. Moreover, correct recognition of tasks as self-chosen involved the right MPFC. Accordingly, the right MPFC may play a role in supporting the maintenance of psychological autonomy and counteract introjection, which individuals with certain personality traits seem to be prone to. This research has significant implications for the study of mechanisms underlying autonomous motivation, goal and norm internalization, decision-making, persuasion, education, and clinical conditions such as depression and burnout.

## Introduction

Is this what I really want? Am I autonomously choosing and pursuing goals or am I simply towing the line of social expectations? Difficulties distinguishing social expectations that may come from parents, partners, teachers, superiors, peer groups, or idols from one’s own goals are especially salient in some individuals. When an expected goal is of low attractiveness (that is, that the individual does not identify with it) but is nonetheless adopted by the person, the process of goal internalization, which typically occurs unconsciously, is called *introjection* (Schafer, 1968; Ryan and Deci, 2000, 2017).

The present study pioneers the neural correlates of introjection, how they differ from those of representations of self-chosen goals, and how they relate to personality traits. Doing so is important because introjection is considered a psychological mechanism underlying unsatisfying decisions, unethical behavior, and persuasion (as compared to conviction), but also reduced motivation and well-being, as well as the development of burnout and depression (Ryan and Deci, 2000; Ryan and Deci, 2017). Accordingly, it is no wonder that the subjective question of leading a life in autonomous versus alienated goal pursuit (i.e., of introjected goals) found entrance in theories of influential thinkers such as Sigmund Freud, Carl Rogers, and Fritz Perls.

We begin by elaborating on the notion of introjection and how its relationships to personality traits have been investigated based on a behavioral paradigm that differentiates one from the other goal differentiation paradigm (e.g., Kuhl and Kazén, 1994). Next, we review relevant literature on brain correlates of self-referential information and goals, and present our hypotheses, particularly on the role of the right MPFC in the representation of self-chosen goals and introjection.

### Goal Introjection

In self-determination theory (Ryan and Deci, 2000, 2017) introjection constitutes a particular and relatively non-autonomous level on the continuum of goal internalization, which reaches from a pole of heteronomy (or other-directedness) to the pole of autonomy (or self-determination). Specifically, an introjected goal is experienced as relatively unattractive to pursue and, accordingly, as self-incongruent (“I should”). An individual who pursues a goal that is not congruent to his or her values or preferences, which is the “true self,” may be called “self-alienated” (Wood et al., 2008; Vess, 2019) or briefly *alienated* in the remainder of this article. By contrast, goals that are experienced as relatively attractive and self-congruent refer to the level of goal identification (“I want”).

Steady inclinations toward low psychological autonomy as reflected in introjection disposes to reduced well-being or even the development of depressive or somatic symptoms (Diefendorff et al., 2000; McGregor et al., 2006; Sheldon, 2014). Meta-analyses have demonstrated that reduced autonomy, for example, reflected in introjection, yields similar effects across individualist and collectivist societies (Yu et al., 2018; Howard et al., 2021; see also Chirkov et al., 2003), demonstrating the cross-cultural applicability and relevance of the concept.

Individuals often introject (unpleasant) goals and values from significant others without conscious awareness; they mistake others’ expectations for self-selected goals. This is how we operationalize introjection in the current study (see also the term *self-infiltration*; Kuhl and Kazén, 1994). Introjection can be considered a particularly insidious form of alienation because unconsciousness obscures the cause of potential lack of motivation and dissatisfaction, and, thus, impedes a conscious decision against a goal (Kuhl and Kazén, 1994; La Guardia, 2009).

It has been argued and demonstrated that a condition that more likely renders introjection is reduced *self-access* (Kuhl and Kazén, 1994; see also Baumann et al., 2017; Quirin et al., 2019; Malekzad et al., 2021; for reviews of this work). Self-access refers to the accessibility of integrated (highly interconnected) self-representations, such as autobiographical memories, semantic self-knowledge, preferences, and motives, together form the *integrative self* (e.g., Kuhl, 2000; Kuhl et al., 2015; Quirin et al., 2019, 2021; see Conway and Pleydell-Pearce, 2000; Schore, 2015, for similar conceptualizations). Self-access is typically reduced by negative affect and stress. This “regressive” process seems to be pronounced in individuals with tendencies toward rumination, as they have difficulties disengaging from negative thoughts and affect (Kuhl, 2000; Kuhl et al., 2021; Quirin and Kuhl, 2022; see Baumann et al., 2017; Quirin et al., 2019, for reviews on extensive evidence).

### Behavioral Studies on Introjection and Personality

Introjection has previously been investigated in behavioral studies using the self-other goal differentiation task (see also Kuhl and Kazén, 1994; Jais et al., 2021), which we also applied in the present functional MRI (fMRI) study. This paradigm measures introjection by the number of tasks that participants misremembered as self-selected but that were imposed by an authority (such as an experimenter or a superior) (Kuhl and Kazén, 1994; Baumann and Kuhl, 2003; Kazén et al., 2003; Quirin and Kuhl, 2018). In these previous studies, introjection was predicted by trait rumination, conceived of as a tendency to be preoccupied with negative thoughts and emotions relating to adverse experiences (Kuhl, 1994; Nolan et al., 1998; Kuhl and Baumann, 2000; Beckmann and Kellmann, 2004; Watkins, 2008; Kröhler and Berti, 2019).

Findings from other studies (Quirin and Kuhl, 2018; Jais et al., 2021) suggest that this relationship might be explained by reduced emotional self-awareness in individuals with rumination tendencies. This is likely because the self-other differentiation task prompts participants toward goal decisions based on how it may feel to work on the task activity (rather than on eventual benefits participants may gather from completing the task – an option that is less likely in the present paradigm). Such a decision requires emotional self-awareness, which can be considered the ability to identify one’s affective reactions (see Taylor et al., 1992; Goleman et al., 2017; Quirin et al., 2021), which is considered a major aspect of self-access (e.g., Quirin and Kuhl, 2018).

### Neural Correlates

So far, the representation of introjected versus correctly remembered self-chosen goals and their relationships with personality traits have not been investigated with neuroimaging techniques. Yet, there is evidence on neural correlates of self-referential information in general, as many studies have investigated, for example, the recognition of one’s face or the self-attribution of own traits (e.g., Gillihan and Farah, 2005; Northoff et al., 2006; Van Overwalle, 2009; Zaki and Ochsner, 2011). Specifically, meta-analyses suggest a role of cortical midline structures in self-referential processing, with the MPFC being particularly involved in representing self-referential information and discriminating them from information referring to others (Northoff and Bermpohl, 2004; Northoff et al., 2006; Qin and Northoff, 2011; Denny et al., 2012).

Particularly, the frontal pole as a subregion of the MPFC has been linked to the representation of goals (Burgess et al., 2011; Cona et al., 2015). However, this research did not distinguish between self-selected goals and those imposed by others. Research using functional near-infrared spectroscopy methodology has shown greater right MPFC activation during personal decision-making in high-difficulty choice situations (Di Domenico et al., 2013), suggesting that a particularly right MPFC activation may promote self-coherence by enhancing the utilization of self-knowledge in the resolution of decisional conflicts. The MPFC also has been shown to be more strongly involved in personal decision-making conflicts when participants’ basic needs were fulfilled (Di Domenico et al., 2013).

Not the least, two behavioral studies applying the self-other goal differentiation paradigm found that an intervention of right hemisphere stimulation by 1-min contralateral (i.e., left) hand contraction engendered reduced introjection rates (Baumann et al., 2005). This is compatible with the authors’ notion that the right, more than the left, MPFC provides access to self-referential representations, including self-chosen goals (Kuhl et al., 2015; Quirin et al., 2019).

Based on this literature, we hypothesized that the right MPFC is involved in the representation of self-chosen goals. As this area should also be recruited in the attempt to distinguish self-chosen goals from social expectations, high introjection rates (in terms of imposed tasks that were falsely self-ascribed) should show reduced right MPFC activity, which is particularly assumed for individuals with a tendency toward rumination and reduced emotional self-awareness. Moreover, as introjection constitutes a conflict (i.e., between self-chosen goals and social expectations), the dorsal ACC, which is known as a conflict monitor and error detector beyond others (Carter et al., 1998), may additionally be activated in the representation of unconsciously introjected goals. Also, besides rumination and emotional self-awareness, we explored potential effects of neuroticism and introversion (i.e., low extraversion), as these factors are linked to many psychological conditions, such as depression and anxiety (Kotov et al., 2010), internalizing problems (Kerber et al., 2021), and rumination tendencies (e.g., Roelofs et al., 2008).

## Materials and Methods

### Participants and Procedure

Data from seventeen German students (mean age = 23.6, range = 19–36 years) were analyzed in the present study. They were recruited *via* e-mail from student communities at the University of Osnabrueck and the University of Bremen in Germany. To warrant homogeneity of the sample with respect to brain functionality (such as hemispheric laterality), we recruited only male and right-handed participants. Moreover, they were all German native speakers, and none had a history of psychological disorders.

**FIGURE 1:**
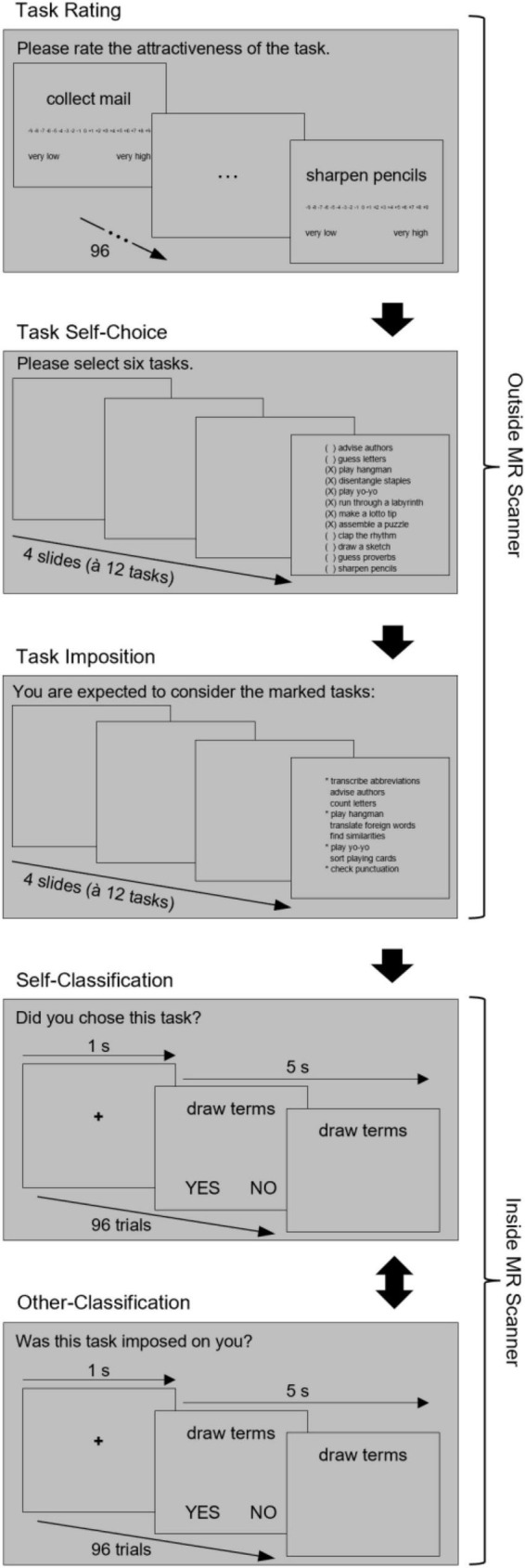
Study design: phases (blocks) of the self-other goal differentiation procedure and their components. While the sequential order of the first three phases was the same for all participants, the last two phases were counterbalanced between participants.

Participants were informed about the study and that it aimed to better understand how people make decisions about what tasks to accomplish throughout a working day. They signed informed consent and filled in a battery of questionnaires including the personality trait scales reported below. Subsequently, participants engaged in the self-other goal differentiation procedure, where they pictured themselves as an employee who has to tackle a subset of tasks from a total list of 96 very different tasks (Kuhl and Kazén, 1994; Baumann and Kuhl, 2003; Kazén et al., 2003; Baumann et al., 2005; Quirin et al., 2009). As in previous research, we made sure that all 96 tasks were of relatively low value (attractiveness) to warrant later introjection of the task goals rather than identification with them. Task value ratings turned out to show a mean of 0.5 and an SD of 3.9, indicating an expected low task value, which was comparable with the literature cited above. Participants were subsequently guided through the five phases of the self-other goal differentiation procedure, which we will describe in the following paragraphs (see also Figure 1).

In the first phase (attractiveness ratings), participants provided attractiveness ratings on 96 tasks (e.g., *sharpening pencils, labeling folders*) presented one by one by using a Likert scale from −9 (*very low*) to +9 (*very high*). In the second phase (self-choice), participants selected 48 tasks from the complete list of 96 tasks for putative later enactment. Before, the computer program split tasks at the median into two groups of very low versus moderately low attractive tasks based on the previous ratings (remind that the tasks were generally of low attractiveness). Four sublists of twelve very low attractive tasks and four lists of twelve moderately low attractive tasks were presented on separate screens from each of which participants were asked to select six (i.e., 48 in total). This way, participants were “forced” to select half of the tasks they previously rated as moderately attractive, but half of the tasks rated as low attractive as well (in order not to confound valence with task source: self vs. other).

In the third phase (task imposition), 48 tasks were assigned to the participant for later (putative) execution by the computer-simulated office superior. Participants were asked to memorize them accordingly. Specifically, tasks were randomly presented in lists of 12 again, with six imposed tasks as indexed by asterisks. Twenty-four of the self-selected tasks were additionally and randomly imposed by the computer-simulated superior. Consequently, 24 tasks remained in the list, that is, they were neither self-selected nor imposed, and, thus, served as control items. For the analyses below, we only used the 24 purely self-selected, the 24 purely imposed, and the 24 remaining items.

In the fourth and fifth phases (source classifications; within the MR scanner), participants unexpectedly judged whether the tasks were: a) self-selected or not (self-choice block), or b) imposed or not (imposition block). In each of the two source memory blocks, the 96 tasks were presented one by one to be able to assess brain activation during these single events. Self-choice and imposition blocks were counterbalanced between participants, the 96 tasks were presented in a random order in either block, and participants provided yes-no answers by pressing a button using their left and right index fingers (counterbalanced between participants). Finally, after having run through the behavioral procedure, which lasted about 60 min, participants were paid and debriefed about the purpose of the study.

### Behavioral Assessment

#### Personality Assessment

We assessed trait rumination (i.e., preoccupation by negative thoughts and emotions that refer to adverse experiences; see Beckmann and Kellmann, 2004) using the preoccupation-subscale of the action control scale (Kuhl, 1994). Two representative example items are: *When I am told that my work has been completely unsatisfactory*, (a) *I don’t let it bother me for too long versus* (b) *I feel paralyzed*, and *When something gets me down*, (a) *I have trouble doing anything at all* versus (b) *I find it easy to distract myself by doing other things*. Rumination-related (“state-oriented”) answers added positively to the rumination score. We measured reduced emotional self-awareness using the difficulties-identifying-feelings subscale of the Toronto Alexithymia Scale comprising seven items (Taylor et al., 1992). Neuroticism and introversion (vs. extraversion) were measured by the NEO Five-Factor Inventory, each comprising twelve items (Costa and McCrae, 1992).

#### Introjection Score

To obtain a behavioral introjection score, we added up the number of false self-ascriptions of imposed tasks and corrected them for the number of falsely self-ascribed remaining tasks to exclude the possibility that potential effects can be attributed to general memory errors (Kuhl and Kazén, 1994; Baumann and Kuhl, 2003; Kazén et al., 2003; Baumann et al., 2005).

### MRI Data Acquisition

Functional whole-brain images were acquired by a Siemens Allegra 3T MRI head scanner (36 slices, TR = 2,000 ms, TE = 30 ms, flip angle = 80°, thickness = 3 mm^3^, and a 65 × 52 matrix yielding a 3 × 3 mm^2^ in-plane resolution), realigned, spatially normalized with a final resolution of 2 × 2 × 2 mm^3^, smoothed with an isotropic 10-mm Gaussian kernel, and analyzed with standard linear regression techniques using the brain imaging software (Statistical Parametric Mapping version 8; Penny et al., 2011). Trials fell into categories (conditions) according to their source (self-selected vs. imposed vs. remaining) and according to participants’ responses about whether tasks were correctly or incorrectly recalled as self-selected or not, or as imposed or not, and were subsequently subjected to an event-related analysis.

### Statistics

Brain imaging data were analyzed using Statistic Parametric Mapping (SPM; version 8) on Matlab (R2014b), whereas behavioral data and their relationships with imaging data were analyzed using Statistical Package for the Social Sciences (SPSS; version 21) and R (version 4.0.5). Conditions of interest were contrasted with (adjusted for) same-block conditions where remaining tasks were correctly refused as being self-chosen or imposed. Following fMRI standards, a significance threshold of *p* < 0.001 (uncorrected) on the voxel level was used for the contrasts. On the cluster level, small-volume Family-Wise-Error (FWE) corrections (Han and Glenn, 2018) were applied for the left versus right MPFC (including orbitofrontal cortices) and the ACC. The significance of hemisphere lateralization was determined by a bootstrap test procedure implemented in SPM LI (Lateralization Index)-Toolbox (Wilke and Lidzba, 2007).

Region labels and brain coordinates were identified by employing the Montreal Neurological Institute (MNI) space utility tool. To correlate behavioral measures with regional activations, eigenvariates from the volume of interests with 10 mm radius spheres were extracted for the contrasts. The center of the spheres were the coordinates of the cluster maximum determined by the respective contrast outcome. We computed bivariate correlations, bias-corrected bootstraps with 10,000 samples, and 95% confidence intervals.

## Results

Attractiveness ratings did not significantly differ among self-chosen, assigned, or remaining tasks in an ANOVA [F = 0, Pr(>F) = 1]. On average, 17.2 (SD = 3.1) of the 24 purely self-chosen tasks were correctly recognized as self-chosen, 15.1 (SD = 2.5) of the 24 purely imposed tasks were correctly recognized as imposed, 7.8 (SD = 3.7) of the imposed tasks were falsely self-ascribed, and 11.7 (SD = 4.9) of the self-chosen tasks were falsely other-ascribed. Moreover, 7.8 (SD = 2.9) of the 24 remaining tasks were falsely ascribed as self-chosen, and 12.5 (SD = 4.3) were falsely ascribed as imposed.

**FIGURE 2:**
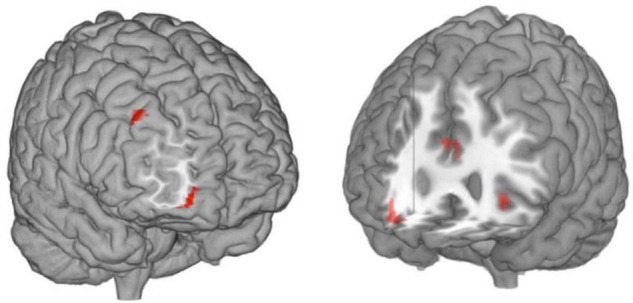
Activation map for self-chosen versus introjected goals. **(Left)** Right MPFC (frontal pole) and dorsolateral PFC response to goals correctly classified as self-chosen. **(Right)** Response of right MPFC, left MPFC, and ACC to introjection.

Table 1 depicts brain activation clusters. As expected, correct recognition of self-chosen tasks involved right MPFC (frontal pole) activation, which was significantly lateralized to the right (LI overall bootstrap-result = −0.36, range = −0.84 to −0.10; see Figure 2, left). In addition, a second cluster was found in the right dorsolateral PFC. By contrast, the correctly recognized imposed tasks were associated with activity in the left MPFC (significant only at voxel level). Introjection in terms of false self-ascriptions of imposed goals (Figure 2, right) involved activation in the right MPFC (*p*_*FWE*_ < 0.05), the left MPFC, and the ACC.

**TABLE 1:** Regions showing BOLD signal changes.

Cluster	Regions	Voxel number	Cluster size	z-Value	Cluster peak			*p*_vox_	*p*_FWE_
					x	y	z		
**Falsely self-ascribed imposed > Falsely self-ascribed remaining tasks (introjection)**									
Right MPFC	R Frontal Pole	83	104	3.90	22	50	−14	<0.001	<0.05
Left MPFC	L Frontal Orbital Cortex		97	4.49	−34	30	0	<0.001	ns
	L Inf. Frontal Gyrus, Pars Triangularis	43							
	L Frontal Operculum Cortex	13							
	L Frontal Pole	3							
	L Insular Cortex	3							
		1							
ACC	Cingulate gyrus, anterior division, and paracingulate gyrus	56	70	3.71	−2	28	24	<0.001	ns
		7							
**Correctly remembered self-chosen > Remaining tasks**									
Right MPFC	R Frontal Pole	22	28	3.98	−24	28	−10	<0.001	ns
**Correctly remembered imposed > Remaining tasks**									
Left MPFC	L Frontal Orbital Cortex	2	9	3.57	20	64	−6	<0.001	<0.05

*p_vox_ = significance threshold at voxel level; p_FWE_ = significance threshold at cluster level (Familywise Error, one-tailed).*

Table 2 shows means, SD, and correlations among activation strength within the right MPFC cluster during introjection, and personality variables. As expected, lower activation in the right MPFC cluster was related to higher introjection rates, self-reports of increased rumination, reduced emotional self-awareness, and increased introversion. In turn, introjection rates were positively related to rumination (replicating earlier behavioral findings: Kuhl and Kazén, 1994; Baumann and Kuhl, 2003; Kazén et al., 2003), as well as to neuroticism and introversion. Applying Cook’s distance criterion, none of these correlations was affected by outliers. Correlations with activity in the left MPFC and the ACC were all non-significant (all p’s > 0.40; not depicted).

**TABLE 2:** Means, SD, and correlations with confidence intervals.

Variable	*M*	*SD*	1	2	3	4	5
(1) rMPFC activation	0.60	0.55					
(2) Introjection Score	−0.47	3.06	−0.53* [−0.81, −0.07]				
(3) Rumination	6.53	2.53	−0.48* [−0.77, −0.02]	0.52* [0.03, 0.79]			
(4) Emotional Self-Awareness	2.30	0.60	0.50* [0.02, 0.79]	−0.29 [−0.68, 0.22]	−0.32 [−0.67, 0.18]		
(5) Neuroticism	2.69	0.62	−0.47 [−0.76, 0.05]	0.63** [0.25, 0.86]	0.56* [0.12, 0.82]	−0.52* [−0.80, −0.05]	
(6) Introversion	2.86	0.59	−0.66** [−0.87, −0.27]	0.59* [0.16, 0.84]	0.43 [−0.06, 0.75]	−0.59* [−0.84, −0.17]	0.59* [0.12, 0.82]

rMPFC, right medial prefrontal cortex; M, mean; SD, standard deviation. Correlation coefficients were averaged across 10,000 bootstraps. Values in square brackets indicate the 95% confidence interval for each correlation based on 10,000 bootstraps. *p < 0.05; **p < 0.01.

## Discussion

The present research brought behavioral research on introjection and its relationship with a personality to a neuroscientific level. We found a strong role of the right MPFC in self-chosen goal representation, as well as reduced activity in this region for introjection and related personality traits, such as rumination tendencies and reduced emotional self-awareness. At the same time, this study extends previous research on neural correlates of self-representations to the subject of self-chosen goals. This previous research revealed a strong role of the MPFC in self-judgments and own face processing (Northoff et al., 2006), and our finding of MPFC involvement in self-chosen goal representation is compatible with these findings. Particularly, the finding of a right MPFC response to own goal representation is compatible with a preponderance of right prefrontal activity for the self during face processing (Northoff et al., 2006) and personal decision-making (Di Domenico et al., 2013), and of a preponderance of right over left MPFC lesions engendering impairments of decision-making, possibly as a consequence of impaired tagging of information related to personal (i.e., self-relevant) decisions with emotional signals (Tranel et al., 2002; D’Argembeau, 2013). The present findings, thus, contribute to the notion that the right MPFC, in particular, may support self-representations or “the self” (Kuhl et al., 2015).

Bilateral MPFC activation for introjected goals suggests that participants activate representations of both their own goals and others’ expectations to check for self-congruence of goals. This may suggest a blurring of boundaries between own goals and others’ expectations. Such checking for self-congruency may amount to a conflict activating the ACC. Speculatively, this may even suggest that, although participants consciously but erroneously think these goals were self-selected, at a low, non-conscious level of behavior regulation, self-incongruence can be detected. However, it is also possible that ACC activation represents low confidence in false as compared to correct judgments about the item source. Accordingly, future research might investigate neural correlates and reaction times of the self-choice of tasks (i.e., decisions) made in the present paradigm and relate them to goal recognition and introjection, as analyzed here.

The finding that low activity in the right MPFC was related to both introjection rates and tendencies toward rumination (and reduced emotional self-awareness) is compatible with behavioral studies suggesting that individuals with a tendency to ruminate engage to a lesser degree in introspectively checking whether a goal was self-selected or not (Kazén et al., 2003). Also comparable with these previous studies are the average numbers of correctly and falsely remembered self-chosen and imposed items.

Whereas behavioral studies conducted in the past investigated relationships between introjection rates and trait rumination (“state orientation”), we additionally examined relationships with neuroticism and introversion and obtained similar results. Although behavioral studies conducted in the past focused on relationships between introjection rates and trait rumination (“state orientation”), in exploratory analyses, we examined relationships with neuroticism and introversion and found similar results. The self-other goal differentiation task was initially named the “process-analytic neuroticism test” (Kuhl and Kazén, 1994) to refer to the original psychoanalytic meaning of “neurotic” in terms of the presence of a conflict between social expectations and personal wishes (“superego conflict”). Likewise, the right MPFC activity was related to these variables, yet non-significantly for neuroticism. Accordingly, future research using the self-other differentiation paradigm may include these personality variables and use hierarchical regression analyses in larger samples to investigate which of the trait variables may most directly (vs. spuriously) be related to introjection. Such analyses would also inform clinical psychology as all self-reported traits are indicative of major psychopathologies, such as anxiety and depression disorders.

Relatedly, the relations found between neural responses to introjection and personality traits are correlational and, thus, cannot be interpreted with respect to causal direction. Previously, it has been theorized that both rumination and introjection may be a consequence of reduced accessibility to the integrative self (i.e., self-access; Quirin and Kuhl, 2018). The right MPFC is considered a central area underlying this self-structure (e.g., Kuhl et al., 2015; Quirin et al., 2019), and its activation, as a causal third variable, may buffer against both rumination and introjection, or even against other maladaptive experiences and behaviors as a possible instantiation of high neuroticism (e.g., anxiety), introversion (e.g., depression), or a combination thereof. This is not in contradiction with the notion that rumination can be elicited by other factors as well.

The present study leaves open whether individuals with a maladaptive personality structure (e.g., high rumination or low self-awareness) have a less developed, integrated self (i.e., non-integrated goals or self-schemata) or whether they just do not have access to it, that is, they suffer difficulties activating it when needed (e.g., Quirin et al., 2021; Quirin and Kuhl, 2022). Previous behavioral studies suggest that the latter might be the case because negative affect and stress have been shown to diminish self-access (Baumann et al., 2017; Quirin et al., 2019, for reviews). Accordingly, to facilitate goal integration and, thus, to reduce introjection, psychological interventions that aim at providing self-access (e.g., in terms of increasing awareness of gut feelings about emotional preferences) may be applied as an alternative to interventions aiming at the long-term development of self-schemata, at least in subclinical cases.

The self-other goal differentiation procedure has been developed to investigate introjection (rather than identification), and consequently draws on tasks of low attractiveness. Accordingly, some readers may question the ecological validity of the findings with respect to self-relevance. First, the self-other goal differentiation task has been validated in many behavioral studies and our current findings conform with the findings of these past studies. Second, free task choice, in general, has been validated as a paradigm to investigate intrinsic motivation and autonomous motives, and the power of having a choice on motivation and psychological health, no matter the valence of the options, has been demonstrated in many studies (Wiechman and Gurland, 2009; Ryan and Deci, 2017). Making a choice among tasks of low attractiveness may function as a proxy for choosing real-life goals (e.g., making a career, or staying healthy) because either case requires self-access in terms of sensing subtle differences in emotional and physical interoceptions.

Also, the study is limited concerning statements about the neural underpinnings of the representation of correctly identified task impositions (as proxies of social expectations or “duties”), which were significant at a cluster level but not when corrected for Family-Wise-Error (FWE). Therefore, future research using larger samples might thoroughly investigate more the neural correlates of social expectations, but also replicate the present findings in general.

Following previous work, between participants, we did not counterbalance the first two phases of the study procedure, as the tasks were only imposed after participants made their choices. Accordingly, potential sequence effects of memory (e.g., enhanced for imposed tasks) cannot be excluded, even if they are not a probable explanation for these results. Nevertheless, future studies may counterbalance the two phases or divide the two blocks of self-selection versus task imposition into several small blocks and present them in a shuffled order to fully rule out these order effects.

This study constitutes a first step in the neuronal underpinnings of the representation of self-chosen goals, social expectations, and their introjection. Particularly, the present work may stimulate neuroscientific research on various psychological phenomena including self-development (becoming a mature, self-determined, and motivated individual), clinical psychology, and interventions (understanding the mechanisms underlying psychological disorders, such as borderline personality disorder, who are considered to have weak self-other boundaries). They are also relevant to questions in philosophy (e.g., addressing questions of “knowing thyself” or the freedom of will), law (e.g., freedom of will as an aspect of liableness and prerequisite of conviction), attitude change (e.g., identifying and complying with political decisions such as COVID-19 regulations), education (e.g., fostering identification over introjection by promoting self-determined choices), economics (e.g., making decisions that are satisfying and in accordance with one’s values and that of the company), or close relationships (e.g., not giving up one’s preferences and identities within a romantic partnership).

## Conclusion

The present study ventured into the neural correlates of self-chosen, imposed, and introjected goals, which is tremendously important to understanding the complex mechanisms underlying human motivation, social behavior, and mental health. Future research is needed to substantiate the present findings and find the psychological and neurobiological levers to foster human autonomy and, thus, diminish alienation as reflected in introjection.

## Data Availability Statement

The raw data supporting the conclusions of this article will be made available by the authors, without undue reservation.

## Ethics Statement

The studies involving human participants were reviewed and approved by the clinical ethics committee of Osnabrück Universit. The patients/participants provided their written informed consent to participate in this study. Written informed consent was obtained from the individual(s) for the publication of any potentially identifiable images or data included in this article.

## Author Contributions

MQ, JK, NB, CK, AK, and MK conceived and designed the experiment. AK collected the data. MQ, AK, RR, ME, and JK outlined the manuscript. AK, MQ, and ER analyzed the data. MQ wrote the manuscript with revisions and contributions from ME, RR, JK, AK, NB, MK, and ER. All authors contributed to the article and approved the submitted version.

## Conflict of Interest

The authors declare that the research was conducted in the absence of any commercial or financial relationships that could be construed as a potential conflict of interest.

## Publisher’s Note

All claims expressed in this article are solely those of the authors and do not necessarily represent those of their affiliated organizations, or those of the publisher, the editors and the reviewers. Any product that may be evaluated in this article, or claim that may be made by its manufacturer, is not guaranteed or endorsed by the publisher.
